# Synthetic Biology and Metabolic Engineering Employing *Escherichia coli* for C2–C6 Bioalcohol Production

**DOI:** 10.3389/fbioe.2020.00710

**Published:** 2020-07-03

**Authors:** Liya Liang, Rongming Liu, Emily F. Freed, Carrie A. Eckert

**Affiliations:** ^1^Renewable and Sustainable Energy Institute, University of Colorado Boulder, Boulder, CO, United States; ^2^National Renewable Energy Laboratory, Golden, CO, United States

**Keywords:** *Escherichia coli*, C2–C6 bioalcohol, ethanol, butanol, isobutanol, isopropanol, isopentanol, isopentenol

## Abstract

Biofuel production from renewable and sustainable resources is playing an increasingly important role within the fuel industry. Among biofuels, bioethanol has been most widely used as an additive for gasoline. Higher alcohols can be blended at a higher volume compared to ethanol and generate lower greenhouse gas (GHG) emissions without a need to change current fuel infrastructures. Thus, these fuels have the potential to replace fossil fuels in support of more environmentally friendly processes. This review summarizes the efforts to enhance bioalcohol production in engineered *Escherichia coli* over the last 5 years and analyzes the current challenges for increasing productivities for industrial applications.

## Introduction

The production of biofuels from renewable resources has gained significant attention due to the rising energy crisis and environmental concerns. Currently, bioethanol is widely used, and Grand View Research, Inc., reported that the global ethanol market size could reach $115.65 billion by 2025, growing at a compound annual growth rate (CAGR) of 6.7%^1^. In addition, the microbial production of higher alcohols (especially C3–C6) has gained traction over the last decade. The use of higher alcohols such as isopropanol or isobutanol would not require changes to current biofuel refinery or transportation processes as these alcohols can be blended at higher volumes in gasoline compared to ethanol (e.g., 16% for isopropanol/isobutanol versus 10% for ethanol), resulting in lower greenhouse gas (GHG) emissions (Andersen et al., 2010; Slating and Kesan, 2012). However, higher alcohols, except n-butanol, are not commonly produced at high yields in microbes. With the development of molecular biology techniques and metabolic engineering strategies, model systems, such as *Escherichia coli* (Jojima et al., 2008; Inokuma et al., 2010; Lan and Liao, 2013; Matsubara et al., 2016) and *Saccharomyces cerevisiae* (Park et al., 2014; Shi et al., 2016), have been modified to synthesize bioalcohols.

**FIGURE 1:**
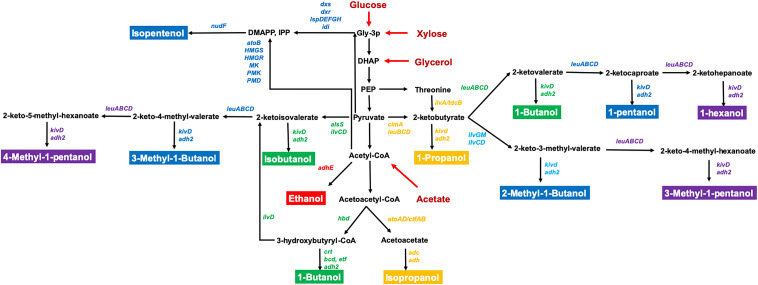
The metabolic pathways for the production of C2–C6 bioalcohol in *E. coli*. The genes directly related to bioalcohol synthesis are labeled in different colors. C2 (red); C3 (orange); C4 (green); C5 (blue); C6 (purple). The substrates are labeled in red. Relevant reactions are represented by the name of the gene(s) coding for the enzyme(s): *adh2/adhE*, alcohol dehydrogenase; *alsS*, acetolactate synthase; *atoB*, acetyl-CoA acyltransferase; *cimA*, citramalate synthase; *dxr*, 1-deoxy-D-xylulose 5-phosphate reductoisomerase; *dxs*, 1-deoxy-D-xylulose 5-phosphate synthase; *HMGR*, 3-hydroxy-3-methylglutaryl-CoA reductase; *HMGS*, 3-hydroxy-3-methylglutaryl-CoA synthase; *idi*, isopentenylpyrophosphate isomerase; *ilvA*, threonine deaminase; *ilvC*, acetohydroxy acid isomeroreductase; *ilvD*, dihydroxy acid dehydratase; *ilvGM*, acetohydroxybutanoate synthase; *ispD*, 4-diphosphocytidyl-2-methylerythritol synthase; *ispE*, 4-diphosphocytidyl-2-methylerythritol kinase; *ispF*, 2-methylerythritol 2,4-cyclodiphosphate synthase; *ispG*, 1-hydroxy-2-methyl-2-(E)-butenyl 4-diphosphate synthase; *ispH*, 1-hydroxy-2-methyl-2-(E)-butenyl 4-diphosphate reductase; *kivd*, ketoisovalerate decarboxylase; *leuA*, 2-isopropylmalate synthase; *leuB*, 3-isopropylmalate dehydrogenase; *leuCD*, 2-isopropylmalate isomerase; *MK*, mevalonate kinase; *nudF*, prenyl phosphatase; *PMD*, phosphomevalonate decarboxylase; *PMK*, phosphomevalonate kinase; *DHAP*, dihydroxyacetone-phosphate; *DMAPP*, dimethylallyl pyrophosphate; *Gly-3-P*, glyceraldehyde-3-phosphate; *IPP*, isopentenyl pyrophosphate; *PEP*, phosphoenolpyruvate.

*Escherichia coli* is a well-studied model microorganism which has several advantageous traits for bioalcohol production including fast growth in inexpensive mineral media, the ability to utilize a wide range of substrates from biomass, and detailed genetic information and diverse genetic tools for gene manipulation. However, there are still challenges using engineered *E. coli* for industrial applications such as the need to improve tolerance to bioalcohols, efficient utilization of low-cost substrates, and productivity toward advanced alcohols (Jojima et al., 2008; Inokuma et al., 2010; Lan and Liao, 2013; Matsubara et al., 2016). Recently, the rapid expansion of genome engineering strategies, synthetic biology techniques, and high-throughput tools have enabled their application to study advanced bioalcohols production and to further investigate the mechanisms of alcohol resistance. This review will summarize recent progress in metabolic engineering of *E*. *coli* for C2–C6 alcohol-derived biofuel production (Figure 1), introduce new synthetic biology methods and genome engineering strategies for in-depth studies of alcohol tolerance, and analyze the current challenges for increasing productivity for industrial applications.

## Ethanol (C2 Bioalcohol) Production in *E. Coli*

The current chassis for industrial ethanol production is *S. cerevisiae* due to its ability to produce ethanol from glucose at 95% maximum theoretical yield (Krishnan et al., 1999). The bacterium *Zymomonas mobilis* has also been proposed for use in industrial ethanol production since it also produces ethanol from glucose at 95% of maximum theoretical yield and has a higher specific ethanol productivity than *S. cerevisiae* (Zhao et al., 2014). Next generation biofuels and biochemicals aim to use lignocellulosic biomass, which contains both glucose and xylose, as an attractive source of non-food carbohydrates for production (Table 1) (Wang L. et al., 2019). However, neither *S. cerevisiae* nor *Z. mobilis* natively utilize xylose. In order to produce bioethanol from cellulosic feedstocks, the cellulose-degrading bacterium *Clostridium thermocellum* has also been used as a chassis for industrial production because it is able to directly ferment cellulose into ethanol. However, *C. thermocellum* only generates ethanol from cellulose (and also only natively utilizes glucose) at 75% of the maximum theoretical yield, resulting in lower ethanol yields compared to *S. cerevisiae* or *Z. mobilis* (Tian et al., 2016). Furthermore, *C. thermocellum* has low tolerance to ethanol (Herrero and Gomez, 1980) and has limited genetic tools, making it difficult and time consuming to engineer this strain for increased ethanol production (Tripathi et al., 2010), although CRISPR-Cas-based genome editing systems were recently developed (Walker et al., 2020).

**TABLE 1:** Select examples of engineering *E. coli* for bioalcohol production.

Alcohol	General strategy	Method	Titer (g/L)	Yield (g/g)	References
Ethanol	Improve substrate utilization	(i) Ethanologenic *E. coli* strains carry the *Z. mobilis pdc*, *adhA*, and *adhE* genes (ii) Deletion of *xylR* gene to make the LYglc1 strain (iii) Deletion of *ptsI*, *ptsG*, *galP*, and *glk*; expression of XylR* to make the LYxyl3 strain (iv) Co-culture of LYglc1 and LYxyl3 strains	46	0.45	Wang L. et al., 2019
Isopropanol	Improve precursor accumulation; decrease the metabolic flux to TCA	Construction of BW25113 (Δ*lacI*, Δ*gltA*) with plasmid pTA17 (*P*_L_lacO_1_::*thl*, *atoAB*, *adc*, and *adhE*), pTA965 (*P*_L_lacO_1_::*tetR*, *P*_L_tetO_1_::*gltA*.LAA), and pTA1251 (*P*_L_lacO_1_::*poxB*, *acs*, *P*lacIq::*lacI*)	3.8	Not reported	Soma et al., 2017
Isobutanol	Directed evolution with alcohol-biosensor-based selection	(i) BmoR-based biosensor used in an atmospheric and room temperature plasma (ARTP) mutagenesis library to screen for increased isobutanol production (ii) Fed-batch fermentation with gas-stripping	56.6	Not reported	Yu et al., 2019
n-butanol	Inactivation of byproduct pathway; improve substrate utilization; improve cofactor supply; adaptive evolution for improved cell growth; optimize the expression of pathway genes	(i) Gene knockout of *hyc-hyp*, *fdhF*, *poxB*, *pck*, *fumB*, *fumAC*, *tdcD*, *mdh*, *focA*, *ppc*, *mgsA*, *yieP*, *stpA*, *yqeG*, *yagM* in BW25113 (ii) Integration of the *fdh* gene into the genome with P_ydfZ_ (iii) Adaptive evolution for fast anaerobic cell growth (iv) RBS library for the *phaA*, *hbd*, *crt*, *ter*, and *adhE2* genes	20	0.34	Dong et al., 2017
2-methyl-1-butanol and 3-methyl-1-butanol	Inactivation of byproduct pathway; improve substrate utilization; optimize the expression of pathway genes	(i) Construction of *E. coli* AY3 (BW25113, Δ*glnA*, Δ*gdhA*, Δ*lsrA*, pYX68 (*ilvE-ilvA-sdaB*), pYX90 (*alsS-ilvC-ilvD-avtA*), pYX97 (leuDH-kivd-yqhD) (ii) Construction of *E. coli* BLF2 [*E. coli* B Δ*ldh*, pLF101 (*alsS-ilvC-ilvD*), pLF102 (*kivd-yqhD*)] (iii) Co-culture of AY3 and BLF2 with an inoculation ratio of 1:4 using distillers’ grains with solubles	2.2 (two alcohols mixture)	Not reported	Liu et al., 2017
Pentanol	Inactivation of byproduct pathway; optimize the expression of pathway genes	(i) Construction of BW25113 (Δ*ilvB* Δ*ilvI* Δ*leuA*) transformed with plasmid pAFC52 (*cimA*Δ2, *leuBCD*) and pGC22 [*leuA* (G462D), *kivd* (V461G), *yqhD*] (ii) *In situ* extraction using oleyl alcohol	4.3	Not reported	Chen et al., 2017
Isoprenol	Improve precursor accumulation; optimize the expression of pathway genes	(i) Construction of AK26 (*E. coli* DH1), transformed with plasmids JBEI-17081 (pA5c-AtoB-HMGS_Sa-HMGR_Sa) and JBEI-17844 (pTrc99a-PMDsc_HKQ-MKmm) (ii) Fed-batch cultures with a solvent overlay	10.8	0.105	Kang et al., 2019

*Escherichia coli* is additionally investigated as a host for ethanol production because it has a large variety of metabolic engineering tools available for strain modification, it can grow in higher concentrations of ethanol (Zaldivar et al., 2000), and it naturally ferments both glucose and xylose, although the presence of glucose still leads to carbon catabolite repression (CCR) when xylose is present (Liu et al., 2018). Thus, Flores et al. (2019) developed an *E. coli* co-culture strategy for conversion of glucose-xylose mixtures to ethanol. One strain, LYglc1, was engineered to only utilize glucose by deleting the xylose-specific transcriptional activator, XylR. The other strain, LYxyl3, was engineered to only utilize xylose by mutating XylR to remove CCR and by deleting genes (Δ*ptsI* Δ*ptsG* Δ*galP glk::kanR*) required for glucose transport and metabolism. The strains are ethanologenic due to insertion of the *pdc*, *adhA*, and *adhE* genes from *Z. mobilis*. The LYglc1 and LYxyl3 strains were co-cultured at an optimum ratio of 1:500, enhancing the sugar utilization rate and ethanol productivity by 50 and 28%, respectively, when compared to a monoculture of the parent strain, LY180. Using this system, they achieved productivity of 0.49 g/L/h, with a final ethanol titer of 46 g/L at 90% of maximum theoretical yield. In a different approach, Sun et al. (2018) constructed a “two-phase-two-temperature” strategy using temperature inducible promoters to control the glucose metabolic pathway in *E. coli*. The final strain, B0013-2021HPA (Δ*ptsG* Δ*manZ* Δ*glk*; *ptsG* expressed under the control of tandem λ pL and pR promoters), utilized all sugars but glucose for cell growth at 34°C, whereas it fermented all sugars to ethanol at 42°C. In addition, *Z. mobilis pdc* and *adhB* genes were introduced to increase ethanol production. As a result, this strain produced 127 g ethanol from 260.9 g mixed sugars from corncob hydrolysate with a productivity of 4.06 g/L/h.

In addition to strategies to improve ethanol yield, there have been several studies to understand ethanol-induced stress and improve ethanol tolerance in *E. coli* (Liu et al., 2016; Cao et al., 2017; Lupino et al., 2018). Cao et al. (2017) systematically analyzed the mechanism for ethanol-induced stress and found that ethanol damages cell wall and membrane integrity, decreases the cross-membrane proton gradient and related ATP synthesis, and changes protein functions by direct binding. Genes that are upregulated in response to ethanol stress include: *osmBC* and *ompCGLR* in response to osmotic stress; *gadABE* and *asr* in response to acid stress; *rpoE*, *degP*, *asnB*, and *opgG* in response to envelope stress; *groSL*, *grpE*, and *metA* in response to heat-shock stress; and the OxyR and SoxRS regulons in response to ROS. Therefore, these cellular processes and genes are good targets for engineering strains with increased ethanol tolerance. Expression of heterologous genes can also lead to increased ethanol tolerance. Liu et al. (2016) expressed the *yajC* gene, which encodes a subunit of a protein translocase complex, from *Lactobacillus buchneri* in *E. coli*, and increased tolerance up to 4% ethanol.

## C3–C4 Alcohol Tolerance and Production in *E. Coli*

C3–C4 alcohols such as propanol, isopropanol, 1-butanol, and isobutanol are higher alcohols which have similar fuel properties (Clomburg and Gonzalez, 2010). Several species of *Clostridium* have been evaluated for butanol and isopropanol production, but cannot be used for industrial application mainly due to low fermentation yield and titer (Survase et al., 2011; Xue and Cheng, 2019). Alternative organisms such as *E. coli* have also been engineered toward the goal of industrial production of C3–C4 alcohols (Jojima et al., 2008; Inokuma et al., 2010; Lan and Liao, 2013; Matsubara et al., 2016). *E. coli* metabolic pathways have shared intermediate metabolites, which reduce central metabolites such as acetyl-CoA and pyruvate into more electron-rich compounds and higher carbon acyl-CoA and 2-keto acids (Saini et al., 2016; Heo et al., 2017; Ohtake et al., 2017; Soma et al., 2017; Nitta et al., 2019).

Precursor accumulation is one of the limiting steps for isopropanol production (Clomburg and Gonzalez, 2010; Soma et al., 2017). The *gltA* gene is involved in synthesizing isocitrate from oxaloacetate. Soma et al. (2014) developed a metabolic toggle switch (MTS) method by expressing the *gltA* gene under the P_*L*_tetO_1_ promoter and the TetR repressor under the P_*L*_lacO_1_ promoter; this system allows metabolic flux from the TCA cycle to be redirected toward isopropanol production in an inducible manner. Then, they introduced a plasmid that overexpressed the native *poxB* and *acs* genes under the P_*L*_lacO_1_ promoter for conversion of excess pyruvate to acetyl-CoA (Soma et al., 2017). The resulting isopropanol titer was up to 3.8 g/L, a titer 4.4-fold higher than that of the parent strain. Our group applied a CRISPR-based genome engineering strategy to generate ribosome binding site (RBS) libraries (903 mutants in total) for genes in the synthetic pathway for the production of isopropanol (*thl*, *atoDA*, *adc*, and *adh*), and were able to identify a high isopropanol producer, PA14, that generated 7.1 g/L at 24 h, with a yield of 0.75 mol/mol glucose. In particular, we found that higher expression levels of *adc* and *adh* led to increased isopropanol production (Liang et al., 2017).

Other efforts have focused on producing bioalcohols from alternate carbon sources. To enable utilization of acetate as the sole carbon source for isopropanol production, Yang et al. (2020) first constructed the isopropanol pathway by combining genes from *Clostridium acetobutylicum* (*thlA*, *adc*), *E. coli* (*atoDA*), and *Clostridium beijerinckii* (*adh*). In addition, they replaced the promoter of the native *ack-pta* genes to improve the acetate kinase and phosphotransacetylase (ACK-PTA) pathway and overexpressed the native *nadK* gene to increase NADH supply. The highest concentration and yield of isopropanol reached was 1.47 g/L and 0.56 g/g acetate. Other efforts have also focused on improving tolerance to isopropanol. Horinouchi et al. (2017) performed adaptive laboratory evolution (ALE) in *E. coli* and identified five mutations (*relA*, *marC*, *proQ*, *yfgO*, and *rraA*) with enhanced isopropanol tolerance up to 27 g/L. Transcriptome analysis revealed that genes related to amino acid biosynthesis, iron homeostasis, and energy metabolisms are related to isopropanol tolerance. Zhou et al. (2019) used a targeted deletion approach and demonstrated that isopropanol tolerance could be increased by inactivation of the acetoacetyl-CoA transferase genes and *atoDA*, enabling growth in 500 mM isopropanol.

Improved fermentative production of isobutanol, a non-native alcohol pathway, has been achieved by metabolic engineering approaches in *E. coli* strains (Blombach and Eikmanns, 2011). Recently, Song et al. (2018) overexpressed the native *acs*, *pckA*, and *maeB* genes to increase acetate uptake, resulting in 26% increased isobutanol titers using acetate as the sole carbon source. Other efforts have focused on increasing isobutanol production from glucose. Liang et al. (2018) introduced the heterologous Entner–Doudoroff (ED) pathway from *Z. mobilis* to increase glucose transformation to pyruvate for enhanced precursor accumulation. The resulting *E. coli* strain, ED02, produced 13.67 g/L isobutanol with a productivity of 0.456 g/L/h, a 56.8 and 88.1% improvement over the parent strain, respectively. Ghosh et al. (2019) used the Optimization by Selection and Sequencing (OptSSeq) strategy to regulate expression levels of genes in the isobutanol synthetic pathway. They found that the optimum levels of pathway enzymes (AlsS, IlvC, IlvD, Kivd, and AdhA) were a molar ratio of 2.5:6.7:2:1:5.2, which led to 3 g/h/gDCW of isobutanol production. In addition, Yu et al. (2019) used a BmoR-based biosensor to screen for improved isobutanol producing strains from an atmospheric and room temperature plasma (ARTP) mutagenesis library. The best isolated variant produced twofold more isobutanol than the wild-type, and the titer of isobutanol reached 56.5 g/L, with a productivity of 0.533 g/L/h, during fed-batch fermentation.

*Clostridium* species have long been employed for n-butanol production through their acetone–butanol–ethanol (ABE) pathway (Xue and Cheng, 2019). However, due to the lack of available genetic tools for *Clostridia*, these species are currently not robust candidates as industrial chassis (Abdelaal et al., 2019). Therefore, in recent years, many efforts have been applied toward engineering *E. coli* for n-butanol production (Dong et al., 2016). Over the last 5 years, the utilization of renewable and cheap substrates from agricultural residues and crude glycerol waste streams have been a target for n-butanol production studies in *E. coli*. Abdelaal et al. (2019) integrated butanol pathway genes (*hbd*, *crt*, *adhE2*, *ter*, and *atoB*) into xylose-utilizing host SSK42 (*E. coli* B P_gapA_PDH Δ*ldhA* Δ*frdA* Δ*pfB*), and the final strain produced 4.3 g/L butanol using xylose as the sole carbon source. Similarly, Saini et al. (2015, 2017) first integrated butanol pathway genes (*phaA*, *hbd*, *crt*, *ter*, and *adhE2*) into a BL21-based host strain with byproduct gene deletions (Δ*ptsG*, Δ*poxB1*, Δ*ldhA*, Δ*frdA*, and Δ*adhE*) for glycerol conversion to butanol. In addition, they increased NADH regeneration by overexpression of genes related to NADH-production (*aceEF*, *lpdA*, *zwf*, *pgl*, and *udhA*). As a result, the engineered strain produced 6.9 g/L n-butanol from 20 g/L crude glycerol under microaerobic conditions, increasing productivity fivefold compared to the strain without modification of NADH supply.

Maintaining cofactor balance and resolving free CoA imbalance are important for CoA-dependent n-butanol production (Nitta et al., 2019). To this end, various groups have overexpressed formate dehydrogenase (Fdh) for NADH regeneration under endogenous fermentation regulatory elements (FREs) control (Wen and Shen, 2016), knocked out the *pgi* gene for increased NADH by activation of the pentose phosphate pathway (PPP) (Saini et al., 2016), decreased carbon flux from acetyl-CoA to the TCA cycle, and improved NADH and CoA supply by downregulation of citrate synthase (Saini et al., 2016; Heo et al., 2017), knocked out genes from the glyoxylate shunt for increased CoA accumulation (Nitta et al., 2019), and optimized AdhE2 activity for CoA recycling and supplemented with cysteine for increased CoA supply (Ohtake et al., 2017). All of these approaches resulted in increased n-butanol production. In addition, Dong et al. (2017) developed a completely chromosomally engineered *E. coli* strain capable of producing butanol efficiently (Table 1). They first integrated the butanol pathway genes into a BW25113-based host strain with deleted byproduct genes. They then modified the expression of *fdh* to increase NADH regeneration and improve anaerobic cell growth by adaptive evolution. The final strain, which also had an optimized butanol pathway (from RBS libraries for pathway genes), produced 20 g/L n-butanol at 83% of theoretical yield, the highest titer achieved compared to the above studies. Low n-butanol tolerance is also limiting for the economic viability of n-butanol production. A number of studies have identified strategies that improve tolerance: (1) mutation of genes related to cis-regulatory elements (*yqjA*, *yabI*, and *rob*) or the efflux pump subunit, acr*B* (Jeong et al., 2017; He et al., 2019); (2) disruption of the transmembrane protein, TqsA (He et al., 2019); (3) disruption of succinylglutamate desuccinylase (AstE) (Guo et al., 2019); (4) overexpression of the chaperone protein SecB or its mutation, SecB_T10A_ (Xu et al., 2019); and (5) overexpression of the membrane-targeted tilapia metallothionein, OmpC-TMT (Chin et al., 2017).

## C5–C6 Alcohol Production and Tolerance in *E. Coli*

C5 alcohols such as 1-pentanol, 2-methyl-1-butanol, and 3-methyl-1-butanol (isopentanol) and C6 alcohols such as 1-hexanol, 3-methyl-1-pentanol, and 4-methyl-1-pentanol represent a useful class of chemicals with potential application as biofuels (Wang et al., 2017). In *E. coli*, iterative keto-acid elongation resulted in C5, C6, and even longer chain (C7–C8) alcohols (Wang et al., 2017). Recently, Eiben et al. (2020) constructed a new isopentanol production pathway in *E. coli* XX03 (BW25113 Δ*adhE* Δ*ldhA* Δ*frdBC*) by introducing the isovaleryl-CoA pathway (LiuC, AibAB, and AibC) from *Myxococcus xanthus* and a butyryl-CoA reductase (AdhE2) from *C. acetobutylicum*, and the resulting strain produced 80.5 mg/L isopentanol after 36 h under microaerobic conditions. In addition, Chen et al. (2017) constructed a 1-pentanol production pathway by controlling the keto acid elongation cycle [BW25113 Δ*ilvB* Δ*ilvI* Δ*leuA* expressing *cimA*Δ2, *leuBCD*, *leuA* (G462D), *kivd* (V461G), *yqhD*] and identifying a new mutation in the ketoisovalerate decarboxylase, KivD V461D, that preferentially tuned the KivD from *Lactococcus lactis* specificity toward 1-pentanol synthesis. The titer of 1-pentanol reached 4.3 g/L and comprised 90% of the total alcohol content. However, the titer, yield, and productivities for C5-C6 bioalcohol production in *E. coli* are still too low for industrial applications. Recently, Chen and coworkers employed an adaptive evolution method to increase the tolerance of *E. coli* to isopropanol, isobutanol, and isopentanol, identifying that upregulated RpoS can increase general alcohol resistance (Wang et al., 2020), a strategy that may also be relevant for tolerance to other C5–C6 alcohols.

The biosynthesis of isopentenols, including isoprenol and prenol, provides an additional route to the production of C5 alcohols (George et al., 2015). Unlike other C5–C6 alcohols, isopentenols are synthesized from the isoprenoid pathway precursor metabolites isopentenyl pyrophosphate (IPP) and dimethylallyl pyrophosphate (DMAPP) (Figure 1). To improve the availability of IPP/DMAPP, Tian et al. (2019) constructed a CRISPRi-mediated multiplex repression system to knockdown genes *asnA*, *gldA*, and *prpE* which are involved in asparagine production, glycerol utilization, and propionyl-CoA synthesis, respectively, resulting in 18-24% higher isopentenol. Kang et al. (2016) developed “IPP-bypass” mevalonate pathways using mevalonate diphosphate decarboxylase (PMD) and phosphatase (AphA) for isopentenol production. They then further improved PMD activity through high-throughput enzyme screening (Kang et al., 2017) and optimized the origin and expression level of genes in this IPP-bypass pathway (Kang et al., 2019). Their final engineered strain had an isoprenol titer of 10.8 g/L in a fed-batch fermentation (Kang et al., 2019). Other efforts have focused on producing isoprenol from cellulosic feedstocks. Wang et al. (2018) initially utilized switchgrass hydrolysate derived from ionic liquid pretreatment for isoprenol production, but the remaining ionic liquids in the hydrolysate required multiple washes to decrease its toxicity to *E. coli*. Thus, they used an adaptive evolution strategy to improve tolerance to ionic liquids, and the adapted *E. coli* strain produced 1.06 g/L which is 6.6-fold more isoprenol compared to the parent strain in the presence of ionic liquids (Wang et al., 2019b). In addition, they found that NaCl enhanced the tolerance of *E. coli* to ionic liquids. MG1655 reached an OD_600_ that was ∼twofold higher with 200 mM NaCl than without NaCl (Wang et al., 2019a).

## Conclusion and Perspectives

Converting renewable biomass into biofuel using engineered microbial cell factories provides a promising alternative to fossil fuels. To date, synthetic pathways have been constructed for the production of bioalcohols ranging from C2 up to C10 (Jang et al., 2012; Kim et al., 2015). The titer, yield, and productivity for C3–C6 bioalcohol production in engineered *E. coli* have improved in recent years; however, the industrial application for the production of these bioalcohols still has some way to go compared to ethanol production. Despite these limitations, some companies (e.g., Butamax and Gevo) have begun to approach commercialization of isobutanol bioproduction. However, additional efforts are still required to overcome technological limitations. New strategies for engineering current microbes still need to be identified or developed for: (1) utilization of non-food-based carbon sources, (2) tolerance to inhibitors derived from biomass or the fermentation process, and (3) tolerance to high concentrations of substrate and biofuel products (Bilal et al., 2018; Shanmugam et al., 2020). While it might be possible to engineer a single microbe for industrial use, microbial consortia could be another solution to improve biofuel production. Cultures containing multiple microbes could improve tolerance to different inhibitors and utilize various components from complex carbon sources, further improving the economics of biofuel production (Bernstein and Carlson, 2012; Shong et al., 2012). In addition, feedstock cost is almost half the cost of the fermentation process (Bhatia et al., 2015), so developing strains that can utilize cheaper feedstocks would have a large economic benefit for industrial biofuel production. Moreover, development of new fermentation processes that increase productivity at the industrial level, such as low-cost and efficient *in situ* product removal (ISPR), could be a new requirement for material and process engineering.

Advances in technologies for DNA synthesis and sequencing have simplified the process of reprogramming metabolism for optimal production of desired chemicals. CRISPR-based technologies have increased the accuracy and speed of gene editing and regulation at genome-scale (Ronda et al., 2015; Garst et al., 2017; Si et al., 2017; Liu R. et al., 2019; Liu Y. et al., 2019). In addition, advanced systems biology tools including genomics, transcriptomics, proteomics, metabolomics, and fluxomics will help facilitate the characterization, analysis, and design of new metabolic pathways for bioalcohol production (Adamczyk and Reed, 2017; McCloskey et al., 2018). Using advanced technologies to gain a deeper understanding of tolerance mechanisms and to refine metabolism for increased titer, yield, and productivity of alcohols from various feedstocks will enable economically viable production of bioalcohols from microbial hosts such as *E. coli*, with findings from *E. coli* providing valuable insight into improving these systems in other non-model microbial chassis.

## Author Contributions

All authors contributed to the analysis of the literature, compiling the related data, and writing the manuscript.

## Conflict of Interest

The authors declare that the research was conducted in the absence of any commercial or financial relationships that could be construed as a potential conflict of interest.

## Footnotes

1 https://www.grandviewresearch.com/press-release/global-fuel-ethanol-market
